# Not dark yet for strong light-matter coupling to accelerate singlet fission dynamics

**DOI:** 10.1016/j.xcrp.2022.100841

**Published:** 2022-04-20

**Authors:** Clàudia Climent, David Casanova, Johannes Feist, Francisco J. Garcia-Vidal

**Affiliations:** 1 Departamento de Física Teórica de la Materia Condensada and Condensed Matter Physics Center (IFIMAC), Universidad Autónoma de Madrid, 28049 Madrid, Spain; 2 Donostia International Physics Centre (DIPC), 20018 Donostia, Euskadi, Spain; 3 IKERBASQUE, Basque Foundation for Science, 48009 Bilbao, Euskadi, Spain; 4 Institute of High Performance Computing, Agency for Science, Technology, and Research (A∗STAR), Connexis, 138632, Singapore

**Keywords:** singlet fission, strong light-matter coupling, polaritons, electronic strong coupling, photophysics

## Abstract

Polaritons are unique hybrid light-matter states that offer an alternative way to manipulate chemical processes. In this work, we show that singlet fission dynamics can be accelerated under strong light-matter coupling. For superexchange-mediated singlet fission, state mixing speeds up the dynamics in cavities when the lower polariton is close in energy to the multiexcitonic state. This effect is more pronounced in non-conventional singlet fission materials in which the energy gap between the bright singlet exciton and the multiexcitonic state is large (>0.1 eV). In this case, the dynamics is dominated by the polaritonic modes and not by the bare-molecule-like dark states, and, additionally, the resonant enhancement due to strong coupling is robust even for energetically broad molecular states. The present results provide a new strategy to expand the range of suitable materials for efficient singlet fission by making use of strong light-matter coupling.

## Highlights

• Singlet fission dynamics is accelerated via polariton formation
• The lower polariton can populate the multiexcitonic TT state
• Cavities can enable efficient singlet fission in materials with large S1-TT gap
• Singlet fission acceleration is robust even for energetically broad molecular states

## Graphical abstract

**Figure:**
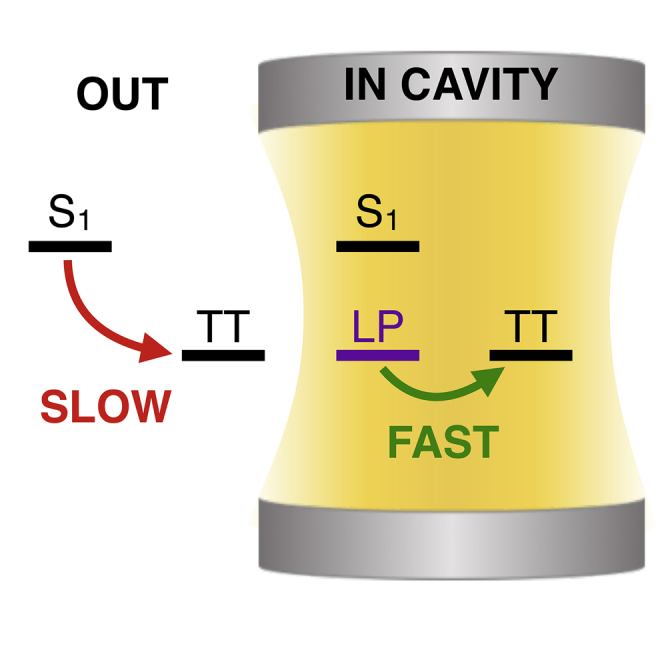

## Teaser

Climent et Al. investigate how the formation of polaritons in optical cavities can affect singlet fission dynamics. Strong light-matter coupling enables efficient singlet fission in materials with large energy gaps between the singlet ($S_{1}$) and the multiexcitonic (TT) state.

## Introduction

The implications that strong light-matter coupling can have in chemistry have recently raised a lot of interest.^1^, ^2^, ^3^, ^4^, ^5^, ^6^ This is because it offers an unconventional way to manipulate chemical processes by modifying the energy landscape as well as the dynamics.^7^^,^^8^ When an ensemble of molecules interacts with a confined light mode, new eigenstates of the system emerge in the strong coupling regime.^2^^,^^6^ This happens when the light-matter interaction exceeds the intrinsic decay rates of both the molecular excitations and the cavity photons. The new eigenstates of the system consist of two hybrid light-matter states known as polaritons and a manifold of dark states, superpositions of the molecular excitations that do not couple to the photon mode. The main difference between both sets of states is that polaritons are delocalized thanks to their cavity photon contribution, while dark states usually behave similarly to uncoupled single-molecule excitons.

In the past decade, there has been a lot of effort devoted to understanding the effects of electronic strong coupling in molecular systems, e.g., the modification of potential energy surfaces,^2^^,^^7^^,^^9^^,^^10^ conical intersections,^11^, ^12^, ^13^, ^14^, ^15^ and electron and energy-transfer phenomena.^16^, ^17^, ^18^, ^19^, ^20^, ^21^, ^22^ The finite lifetime of the cavity photons^23^, ^24^, ^25^, ^26^ and the presence of a dense dark-state manifold^27^, ^28^, ^29^, ^30^, ^31^, ^32^, ^33^, ^34^, ^35^ are key to understanding polaritonic chemistry phenomena. As already noted, dark states may wash out polaritonic effects in setups with collective, i.e., many-molecule, strong coupling, as there is a macroscopic number of them compared with only two polaritons per cavity mode.^36^, ^37^, ^38^ For instance, there is certain controversy on strong light-matter coupling effects on thermally activated delayed fluorescence (TADF), in which a triplet state repopulates the singlet state responsible for the delayed emission.^39^^,^^40^ Some authors have suggested that there are limitations on polaritonic effects on reverse intersystem crossing.^36^^,^^41^ This is because the initial state is localized on a single molecule while a polariton is delocalized over the entire molecular ensemble that couples to the cavity photon. As further discussed below, the rate for population relaxation from a localized to a delocalized state is penalized by a factor 1/N, where *N* is the number of entities participating in the delocalized state.^27^^,^^32^^,^^42^^,^^43^ In polariton-assisted TADF, direct population transfer from the triplet state to the lower polariton (LP) then competes with the much faster process of relaxation between localized single-molecule states; that is, from the triplet state to the singlet dark-state manifold.^36^^,^^41^ However, the reverse process is not penalized when there is a manifold of *N* localized final states, as the factor 1/N in the rate to any single state is compensated by the number of states *N*.

Singlet fission is a downconversion photophysical reaction in which a spin-singlet exciton splits into two independent spin-triplet states (Equation 1).^44^, ^45^, ^46^ The recent interest in this unique phenomenon has been driven by its potential capacity to overcome the Shockley-Queisser limit^47^ for the efficiency of single junction solar cells.^48^, ^49^, ^50^ It is well accepted that the singlet fission process involves the generation of a multiexcitonic intermediate, the zero-spin triplet-pair state (TT),^51^^,^^52^ which eventually splits into two uncoupled triplet states. Commonly, the formation of the TT state (first step in Equation 1) is the rate-limiting process in singlet fission and is thus the main subject of study in the field.

(Equation 1) $$S_{1}\overset{\text{step}1}{⇌}TT\overset{\text{step}2}{⇌}T_{1}+T_{1}$$

In contrast to TADF under strong coupling, a polaritonic mode could potentially be the initial state instead of the final one in a singlet fission process. Previous theoretical works investigating singlet fission under strong coupling have mainly focused on the single-molecule case,^53^, ^54^, ^55^ while studies of the collective situation are still scarce.^42^ There are no clear guidelines yet on if, how, and when singlet fission can benefit from the presence of polaritons. This is the main motivation for the current work exploring cavity-modified singlet fission. We focus on the situation of many-molecule strong coupling, which is experimentally much easier to achieve than few-molecule strong coupling (only possible in strongly subwavelength plasmonic nanocavities^56^), and is simultaneously more promising for leading to practical light-harvesting devices in the strong coupling regime.^57^, ^58^, ^59^, ^60^ On the experimental side, there has been some initial work investigating singlet fission or related processes such as triplet-triplet annihilation in optical cavities or with plasmonic nanostructures.^37^^,^^54^^,^^61^, ^62^, ^63^ Most of them have focused on the long-time (nanoseconds to microseconds) dynamics.^61^^,^^62^ In a recent study, transient optical spectroscopy was employed to monitor the early dynamics of a TIPS-pentacene film placed in a Fabry-Perot resonator.^37^ The rate constants extracted from fitting the experimental data did not show significant differences between cavity and non-cavity situations. These results suggested that the dark-state manifold dominated the dynamics and thus collective strong coupling was unable to modify the singlet fission process.

In this work, we focus on singlet-exciton fission dynamics under collective strong coupling and explore how the dynamics is affected by the presence of dark states and state broadening due to both the natural linewidth of the vibronic peaks and energetic disorder. The central question we aim to answer is whether the state mixing induced by strong light-matter coupling can enhance the singlet fission rate in prototypical organic materials, and which materials are most suitable for such an application. Our results show that singlet fission indeed becomes faster when the LP is spectrally tuned to be on resonance with the multiexcitonic state. Importantly, this mechanism is also operative for non-conventional singlet fission materials that present a large singlet-multiexcitonic state gap. We also find that the enhancement mechanism is not significantly affected by dark-state-induced dephasing in these compounds. Moreover, when energetic state broadening is considered, the enhancement in the rate is more robust against disorder for strongly exothermic materials than for conventional ones. This combination of properties opens up a whole range of opportunities for materials that have not been explored for singlet fission to date. Throughout the manuscript, we use italic characters to denote diabatic electronic states, while eigenstates of the system are indicated in boldface and labeled according to their main diabatic contribution.

## Results and discussion

### Singlet fission dynamics in an optical cavity

To address the feasibility of singlet fission under strong light-matter coupling, we first treat a single-molecule model and then extend it to the many-molecule case in the following section. Our model describes several molecular electronic excitations, a bath of intramolecular vibrations, and a cavity photon. We fully consider the coupling between the electronic excitations and the photonic mode, and treat the electronic-vibrational interaction perturbatively by relying on a master equation approach based on Bloch-Redfield theory. There are two main reasons behind this choice. First, it allows us to naturally incorporate two essential ingredients that have a leading role in light-matter strong coupling: the effect of many molecules as well as the finite cavity lifetime (through an additional Lindblad term). Second, in contrast to the commonly employed Fermi-Golden rule approaches that rely on a perturbative treatment of the electronic couplings,^64^, ^65^, ^66^, ^67^, ^68^ Bloch-Redfield theory treats them exactly and can properly describe singlet fission dynamics when the coupling to the vibrational bath is not too strong.^69^, ^70^, ^71^ Note that the physics of the dark polaritons described in the Holstein-Tavis-Cummings model commonly used to describe molecular polaritons,^29^^,^^30^ which treats a single vibrational mode explicitly, are also represented in our Bloch-Redfield simulations in a slightly different form. For instance, X-type polaritons, which are effectively dark states in the Holstein-Tavis-Cummings model, can be represented within the single-excitation subspace by considering energetic broadening of the states.

The Bloch-Redfield master equation for the time evolution of the density matrix describing the electronic and photonic degrees of freedom can be written as:

(Equation 2) $$\frac{d}{dt}\;\rho_{ab}\left(t\right)=-i\omega_{ab}\rho_{ab}\left(t\right)+\sum_{c,d}R_{abcd}\rho_{cd}\left(t\right)$$

where a,b,c, and *d* indices run over the eigenstates of the system Hamiltonian, $\omega_{ab}$ are the eigenfrequency differences, and $R_{abcd}$ is the Bloch-Redfield tensor accounting for the system-bath interaction; i.e., the coupling between electronic and vibrational modes. Relaxation rates are expressed in terms of the spectral density representing a bilinear electron-phonon interaction that we approximate by an Ohmic function with a Lorentz-Drude cutoff,^69^^,^^71^ for which we have chosen characteristic parameters of representative singlet fission materials. Although the specifics of the spectral density of the environment can be relevant for quantitative predictions for a given molecular species, here we focus on providing insight and general guidelines on the circumstances under which collective strong coupling modifies the singlet fission dynamics, and thus use a general molecule-independent spectral density. Note that we have checked that our results are not strongly affected by the spectral density parameters; that is, the cutoff and reorganization energy.

The system Hamiltonian for the molecule interacting with the cavity mode is given by

(Equation 3) $${\hat{H}}_{S}={\hat{H}}_{el}+{\hat{H}}_{cav}+{\hat{H}}_{el-cav},$$

with the electronic Hamiltonian

(Equation 4) $${\hat{H}}_{el}=\sum_{i}E_{i}\left|i\right\rangle \left\langle i\right|+\sum_{i\neq j}V_{ij}\left|i\right\rangle \left\langle j\right|,$$

where $E_{i}$ and $V_{ij}$ are the energies and interstate couplings, respectively, for the (diabatic) electronic states involved in singlet fission. We use a four-state model to represent a system with the ground state ($S_{0}$), the optically active singlet exciton ($S_{1}$), the triplet-pair state (TT), and a charge-transfer (CT) state, as suggested by Reichman and coworkers.^69^ Specifically, we consider first the case of a slightly exothermic formation of the TT state ($E_{S_{1}}-E_{TT}=80$ meV, $E_{TT}-E_{S_{0}}=1.7$ eV) and a higher-lying CT state ($E_{CT}-E_{TT}=330$ meV). These energetics are representative of efficient singlet fission compounds in which the CT state mediates the population transfer from the $S_{1}$ to the multiexcitonic TT state via a superexchange mechanism.^69^ In many singlet fission materials, the couplings between the CT and the $S_{1}$ and TT states are (at least) one order of magnitude larger than the direct $S_{1}/TT$ interaction, since the former contain one-electron terms while the latter can be approximated as the difference between two bielectronic integrals.^45^ Here we take $V_{S_{1},CT}=V_{TT,CT}=30$ meV, whereas we disregard the direct coupling ($V_{S_{1},TT}=0$ meV). Note that previous work on singlet fission under collective strong coupling focused on a direct mechanism where no CT intermediates are involved.^42^

The cavity term in (Equation 3) takes the form ${\hat{H}}_{cav}=\hbar \omega_{c}{\hat{a}}^{†}\hat{a}$, where $\omega_{c}$ is the frequency of the cavity mode, which we take in resonance with the optical exciton ($\hbar \omega_{c}=E_{S_{1}}-E_{S_{0}}$), and ${\hat{a}}^{†}$ and $\hat{a}$ are the bosonic creation and annihilation operators, respectively. Finally, the interaction between the cavity photon and the electronic states can be expressed by the Jaynes-Cummings Hamiltonian^72^ for the case of a single singlet fission site (N=1):

(Equation 5) $${\hat{H}}_{el-cav}=\sum_{i}\hbar g_{i}\left({\hat{a}}^{†}\left|S_{0}\right\rangle \left\langle i\right|+\hat{a}\left|i\right\rangle \left\langle S_{0}\right|\right),$$

with $g_{i}$ being the coupling strength between the cavity photon and the *i*-th electronic excited state, which is half the Rabi frequency (${\text{Ω}}_{R}$). Unless otherwise indicated, we consider strong coupling to the bright state, with $g_{S_{1}}=75$ meV. We precisely choose this value because it is roughly equal to the energy gap between the $S_{1}$ and TT states, thus placing the LP close to the TT eigenstate. On the other hand, we consider both TT and CT to be optically non-active transitions, hence, their coupling to the cavity vanishes. In this work we are interested in the linear-response regime, such that simulations can be restricted up to the single-excitation subspace.

We account for the finite lifetime of the cavity photon due to radiative and nonradiative decay by including a Lindblad term in our simulations. In this work, we have considered a 50-ps cavity lifetime. We have checked that the minimum cavity lifetime necessary for our results to hold is around 10 ps. Note that, for cavity photon frequencies around 2 eV, as those we consider here, this would correspond to a quality factor of $Q∼3\cdot 10^{4}$. While such Q factors are beyond those used in the context of molecular strong coupling, we note that they can be achieved experimentally at optical frequencies.^73^^,^^74^ In particular, in Najer et al.,^74^ a value of $Q∼10^{5}$ was reported.

We would like to stress that, when planning to manipulate molecular photophysics with cavities, it is important to be aware of this additional deactivation channel, which is absent in the non-cavity situation, and might compete with the intrinsic molecular processes. In the situation we explore in this work, the non-cavity singlet fission rate is faster than the cavity decay. Bearing in mind that TT formation in pentacene, one of the most efficient singlet fission materials, occurs within 80 fs,^75^ one should aim for cavity lifetimes beyond the femtosecond range in order to modify singlet fission dynamics via polariton formation.

**Figure 1:**
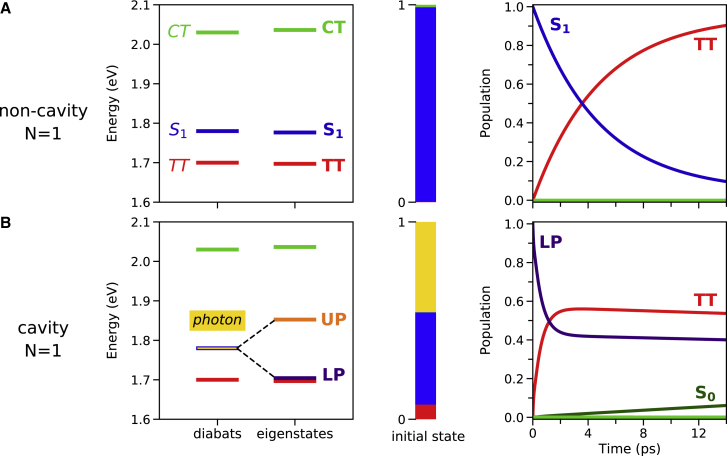
Energy spectra and population dynamics for N = 1 (A and B) Energy spectrum (left), initial eigenstate diabatic composition (middle), and population dynamics (right) for (A) bare N=1 case, and strong coupling with (B) N=1 ($g_{S_{1}}=75$ meV). Note that the eigenstates are colored according to their main diabatic contribution. Color code: cavity photon |1⟩ (yellow), $S_{0}$ (dark green), TT (red), $S_{1}$ (dark blue), CT (lime green), LP (indigo), and UP (orange).

The singlet fission dynamics within and outside the optical cavity is represented in Figure 1. For the non-cavity case (Figure 1A), the initially populated $S_{1}$ state relaxes to the TT state with a mean time of 5 ps, as obtained from the Bloch-Redfield rate from the adiabatic $S_{1}$ to the TT eigenstate,

(Equation 6) $$k_{S_{1}\to \mathrm{TT}}=\left(\alpha_{TT}^{2}\beta_{TT}^{2}+\alpha_{S_{1}}^{2}\beta_{S_{1}}^{2}+\alpha_{CT}^{2}\beta_{CT}^{2}\right)S\left(\omega_{S_{1},\mathrm{TT}}\right),$$

expressed in terms of the eigenstate expansion coefficients $\left|S_{1}\right\rangle =\sum_{i}\alpha_{i}\left|i\right\rangle$ and $\left|TT\right\rangle =\sum_{i}\beta_{i}\left|i\right\rangle$, and the power spectrum S(ω), which characterizes the environment’s ability to absorb or release the energy required for the transition between the two eigenstates to happen at a given finite temperature (in our case, room temperature). According to this expression, the singlet fission rate will be non-zero under two conditions: (1) both states must have a common diabatic contribution, and (2) the environment of the molecular vibrations is able to meet the energetic requirements for the transition to occur. As shown in Figure 1A, the very small TT and CT contributions to the adiabatic $S_{1}$ state are sufficient to drive the population relaxation. Note that the time evolution of the photophysical reaction proceeds with no significant population of the CT state, which acts only as a mediator agent.

Next, we focus on how strong light-matter coupling can affect the singlet fission dynamics. In this case, we explore the time evolution of the system starting from the LP. Since the LP→TT population transfer rate is analogous to that for the non-cavity case in Equation 6 by replacing $S_{1}$ with LP, then a straightforward strategy to enhance the singlet fission rate might be to increase the mixing between states. For that, we couple the cavity photon with the $S_{1}$ state such that the LP is placed close to resonance with the TT state. In this scenario, the LP acquires some TT character and the TT state acquires some polaritonic, i.e., $S_{1}$ and cavity photon, character, thus enhancing the LP→TT population transfer rate. This is indeed what happens, as shown in Figure 1B, where the singlet fission dynamics is accelerated at earlier times compared with the $S_{1}\to TT$ non-cavity situation, with a considerably shorter mean time (0.4 ps). Note that state mixing was invoked in Polak et al.^62^ to qualitatively interpret the reverse process, triplet-triplet annihilation, in an optical cavity. Although the rate populating the TT state is modified under strong coupling, the CT-mediated singlet fission mechanism is maintained, as shown by the nearly null population of the CT state during the entire photophysical reaction, and the fact that the multiexcitonic state is not populated without the presence of the CT state. Notice also that, because of the finite cavity lifetime (50 ps), the ground state ($S_{0}$) becomes populated due to the non-zero cavity photon character of the LP and TT states. It is important to highlight that, since the LP is practically degenerate with the TT state, it is pure dephasing that promotes the population transfer; i.e., it is the bath spectral density evaluated at zero frequency that determines the Bloch-Redfield rate and, therefore, energy-dependent details in this spectral density are not too critical here. Finally, note that although we have not taken into account the cavity dispersion in our simulations, it could be detrimental to the polariton-accelerated singlet fission rate we observe because an additional relaxation channel, namely, intra-band scattering in the LP branch, would be present.

### Collective strong light-matter coupling

In the following we extend our model to the case of *N* equivalent (non-interacting) singlet fission sites, that is, multiple $\left\{S_{1},TT,CT\right\}$ electronic systems, interacting with a cavity photon. For that, we expand the electronic terms of the system Hamiltonian following the so-called Tavis-Cummings model:^76^^,^^77^

(Equation 7) $${\hat{H}}_{el}=\overset{N}{\underset{k=1}{\sum }}\left[\sum_{i}E_{i}\left|i_{k}\right\rangle \left\langle i_{k}\right|+\sum_{i\neq j}V_{ij}\left|i_{k}\right\rangle \left\langle j_{k}\right|\right],$$

(Equation 8) $${\hat{H}}_{el-cav}=\sum_{i}\hbar g_{i}^{\left(N\right)}\sum_{k=1}^{N}\left({\hat{a}}^{†}\left|S_{0_{k}}\right\rangle \left\langle i_{k}\right|+\hat{a}\left|i_{k}\right\rangle \left\langle S_{0_{k}}\right|\right),$$

where $\left|i_{k}\right\rangle$ corresponds to an electronic excitation ($TT,S_{1}$ or CT) at the *k*-th singlet fission site, and the light-matter coupling strength is the same for all *k* sites and is chosen so that the collective Rabi splitting ${\text{Ω}}_{R}$ stays constant ($g_{i}^{\left(N\right)}=\delta_{iS_{1}}{\text{Ω}}_{R}/\sqrt{4N}$). In the following we also restrict the system to one excitation at most. Note that we do not consider permanent dipole moments in our model since we are interested in the collective regime where these are not enhanced by a $\sqrt{N}$ factor, as opposed to the transition dipole moment. In the following, we show results for N=20. Note that the relevant parameter here is the collective Rabi splitting and that we have checked that our results are converged for this value.

**Figure 2:**
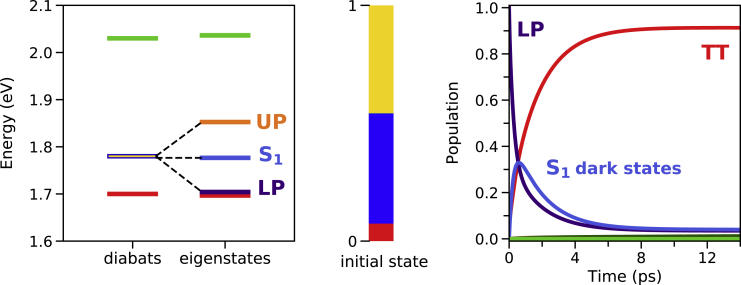
Energy spectra and population dynamics for N = 20 Energy spectrum (left), initial eigenstate diabatic composition (middle), and population dynamics (right) for N=20 ($g_{S_{1}}=17$ meV). Note that the eigenstates are colored according to their main diabatic contribution. Color code: cavity photon |1⟩ (yellow), $S_{0}$ (dark green), TT (red), $S_{1}$ (dark blue), CT (lime green), LP (indigo), UP (orange), and $S_{1}$ dark states (royal blue).

The most important difference between the single singlet fission site previously discussed and the collective case is the presence of the dark singlet-exciton manifold; i.e., linear combinations of the molecular $S_{1}$ states that do not couple to the cavity photon. These states, together with the two polaritons and the TT and CT manifolds, constitute the eigenstates of the collective system. As shown in Figure 2, the fast LP→TT relaxation is maintained, and because of detailed balance, the TT saturation population increases since for larger *N* the many TT states outnumber the LP and act as a population sink. Therefore, this polariton-enhanced mechanism does not suffer from the issues arising in polariton-assisted TADF^36^^,^^39^ previously discussed, in which a single-molecule excitation has to transfer to a collective polariton, incurring a 1/N penalty in the transition rate.

It is straightforward to understand why the rapid initial relaxation from the LP to the TT manifold still holds in the collective case. The collective strong coupling version of Equation 6 is given by

(Equation 9) $$k_{LP\to TT_{q}}=\left(\sum_{k}^{N}\alpha_{q,TT_{k}}^{2}\beta_{TT_{k}}^{2}+\alpha_{q,S_{1_{k}}}^{2}\beta_{S_{1_{k}}}^{2}+\alpha_{q,CT_{k}}^{2}\beta_{CT_{k}}^{2}\right)S\left(\omega_{LP,TT_{q}}\right),$$

where q=1…N labels the specific state of the TT manifold. These states are basically linear combinations of the non-cavity TT eigenstate on each site, $\left|TT_{q}\right\rangle \approx \sum_{k}^{N}\alpha_{q,TT_{k}}\left|TT_{k}\right\rangle$, therefore, the rate constant can be approximated as $k_{LP\to TT_{q}}\approx \sum_{k}^{N}\alpha_{q,TT_{k}}^{2}\beta_{TT_{k}}^{2}S\left(\omega_{LP,TT_{q}}\right)$. Since the LP amplitude of the TT state on each *k*-site is equal to the N=1 amplitude scaled by 1/N, i.e., $\beta_{TT_{k}}=\beta_{TT}/\sqrt{N}$, and since $\sum_{k}^{N}\alpha_{q,TT_{k}}^{2}\approx 1$, then $k_{LP\to TT_{q}}\approx \beta_{TT}^{2}S\left(\omega_{LP,TT_{q}}\right)/N$. Therefore, in the macroscopic limit, the rate from the LP to the set of *N* $\left\{TT_{q}\right\}$ states is independent of *N* and is dictated by the amount of TT character the LP acquires in the N=1 case ($\beta_{TT}$). According to this analysis, collective strong coupling will affect the early singlet fission dynamics when the LP is close to resonance with the TT manifold, such that $\beta_{TT}\neq 0$. Notice that, in the collective case, the ground state is barely populated, in contrast to the single-site results (Figure 1B). This is because only one eigenstate of the TT manifold has a non-vanishing cavity contribution.

### Promoting strongly exothermic singlet fission

An important factor at play here that we have not addressed yet is the role of the (N−1) {$S_{1q}$} dark states. In the example we have discussed, since this manifold is relatively close in energy to the LP, it is significantly populated during the dynamics, as shown in Figure 2. From the $S_{1}$ dark states, singlet fission then proceeds with essentially the same rate as in the non-cavity situation, then diminishing the rate enhancement due to strong coupling. However, the further these {$S_{1q}$} dark states are spectrally separated from the LP and the TT manifold, the less populated they will be after excitation to the LP and, as a consequence, they will be less detrimental to the cavity-based singlet fission rate enhancement. Therefore, we hypothesize that the ideal candidates for singlet fission under collective strong coupling are those compounds with a large enough $S_{1}-TT$ gap, such that, when the LP is excited, the {$S_{1q}$} dark states are energetically too high to be populated. Standard singlet fission materials exhibit an $S_{1}$ state that ideally lies slightly above the multiexcitonic TT singlet state, i.e., weakly exothermic singlet fission, which severely limits the pool of potentially efficient singlet fission compounds. Also, prototypical efficient singlet fission materials, such as pentacene and rubrene, have poor photochemical stabilities,^78^, ^79^, ^80^ preventing their use in real-world devices. Therefore, according to our hypothesis, strong coupling could enhance the singlet fission rate in materials that have usually been ignored since excessive exoergicity is known to be detrimental in the non-cavity situation.^44^^,^^81^^,^^82^

**Figure 3:**
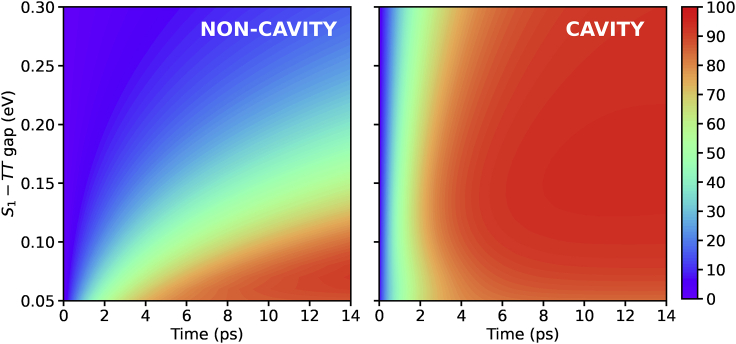
Adiabatic %TT population for the non-cavity ($S_{1}$ initial state) and cavity (LP initial state and N=20) situations The single-molecule coupling constant $g_{S_{1}}=10-66$ meV as the $S_{1}-TT$ gap increases since a larger Rabi splitting is needed for the LP to be close to resonance with the TT eigenstates.

To test our hypothesis, in Figure 3 we plot the TT population dynamics dependence on the diabatic $S_{1}-TT$ gap while keeping the $CT-S_{1}$ gap constant. For the cavity case, as the $S_{1}-TT$ gap varies, the Rabi splitting is chosen such that the LP lies close to resonance with the TT eigenstates (5−10 meV above), just like in Figures 1B and 2. For the non-cavity case, the singlet fission dynamics is greatly slowed down as the separation between the two states increases as the amplitude contribution in Equation 6 decreases due to reduced state mixing. In contrast, within the cavity, the TT state is populated much faster and independently of the $S_{1}-TT$ gap, as long as the LP is brought close to resonance with the TT eigenstates. These results therefore indicate that strong light-matter coupling can also accelerate the singlet fission dynamics of materials with large $S_{1}-TT$ gaps.

**Figure 4:**
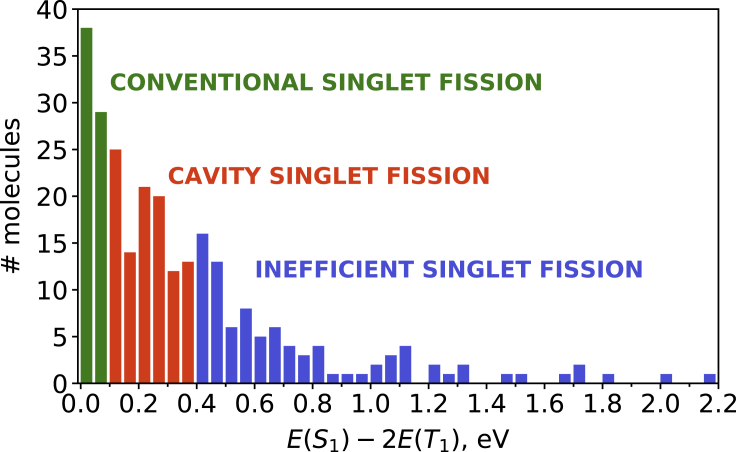
Histogram (in eV) of a list of 262 organic molecules Experimental structures from Montalti et al.^88^ Singlet and triplet energies obtained from Padula et al.^84^

Therefore, within the strong coupling regime, it becomes possible to efficiently generate triplet-pair states not only for slightly exothermic energetics; e.g., in pentacene. Relaxing the near-degeneracy criterion ($E\left(S_{1}\right)-2E\left(T_{1}\right)∼0$) should allow fast singlet fission processes to be obtained in a wider range of molecular systems with respect to those identified to date. In order to exemplify the potential impact in the search for singlet fission chromophores, in the following, we consider a set of organic molecules with suitable properties: (1) exothermic singlet fission ($E\left(S_{1}\right)-2E\left(T_{1}\right)>0$); (2) non-vanishing transition dipole moment between ground and lowest excited singlet, so strong coupling to the cavity mode could be achieved; and (3) sufficiently large (>0.4 eV) $S_{0}$-to-$T_{1}$ gap in order to ensure molecular stability for practical applications. Our molecular test set contains 262 organic molecules obtained from the Cambridge Structural Database (CSD)^83^ selected following the protocol designed by Padula and collaborators.^84^ In order for singlet fission to compete with other relaxation pathways, such as internal conversion, intersystem crossing, or radiationless decay to the ground state, it should take place on a picosecond (or even sub-picosecond) timescale. Therefore, here we consider that good candidates for efficient singlet fission are those able to reach a 50% population of the TT state within 5 ps after photoexcitation. Of course, this is a somewhat arbitrary limit, but it reasonably serves our purpose to compare singlet fission efficiency with and without strong coupling. For the non-cavity scenario, it occurs for $E\left(S_{1}\right)-E\left(TT\right)\leq 0.1$ eV (Figure 3), which is in the order of the gap measured in crystalline pentacene,^85^^,^^86^ and slightly higher than the value employed in the previous sections (0.08 eV). About 26% of the considered chromophores exhibit $E\left(S_{1}\right)-E\left(TT\right)\leq 0.1$ eV (green bars in Figure 4) and are expected to be the most promising singlet fission compounds. In contrast, if we consider ∼0.8 eV as the upper limit for the Rabi splitting,^8^^,^^87^ the number of potential molecules able to undergo singlet fission efficiently when placed in an optical cavity increases to 66% of the total set of studied molecules (orange bars in Figure 4). These results demonstrate how strong coupling can substantially increase the pool of suitable molecular candidates, which represents one of the main current challenges for the practical implementation of singlet fission in optoelectronic devices.^44^ Also note that the values reported here can be seen as rather conservative, since in the cavity model the $S_{1}-CT$ energy difference remains constant while varying the $S_{1}-TT$ gap, debilitating the efficiency of the CT-mediated mechanism (for large $S_{1}-TT$ gaps, the CT states lie rather high with respect to LP and TT). Moreover, we have not considered here the case of direct singlet fission, i.e., direct $S_{1}/TT$ coupling, which would also benefit from the energy-level alignment between LP and TT states.

### Effect of state broadening

So far we have assumed equivalent singlet fission sites with well-defined discrete TT, $S_{1}$, and CT energies. Since our conclusions rely on the LP being close to resonance with the TT manifold, it is not clear whether they will still hold when taking into account the energetic broadening of the states. In the following, we account for the linewidth of the Franck-Condon vibronic transition that couples to the cavity mode and also inhomogeneous broadening due to energetic disorder because of different local environments. To explore this effect, we select the molecular energies by sampling a Gaussian distribution centered at $E_{i}$ with $i=TT,S_{1},CT$. We consider a maximum full-width half-maximum (FWHM) value of 0.1 eV, which is characteristic of the $S_{1}$ vibronic absorption peaks of polycyclic aromatic hydrocarbons.^89^ We explore two limiting cases, one in which the energies of the three molecular states of each singlet fission site are jointly sampled, that is, the same random number is used to sample the Gaussian distribution for all three states of a given site, and another one for which the three states are sampled independently by using different random numbers. Note that, in the former case, the energy gaps between states remain constant, while in the latter they may vary.

**Figure 5:**
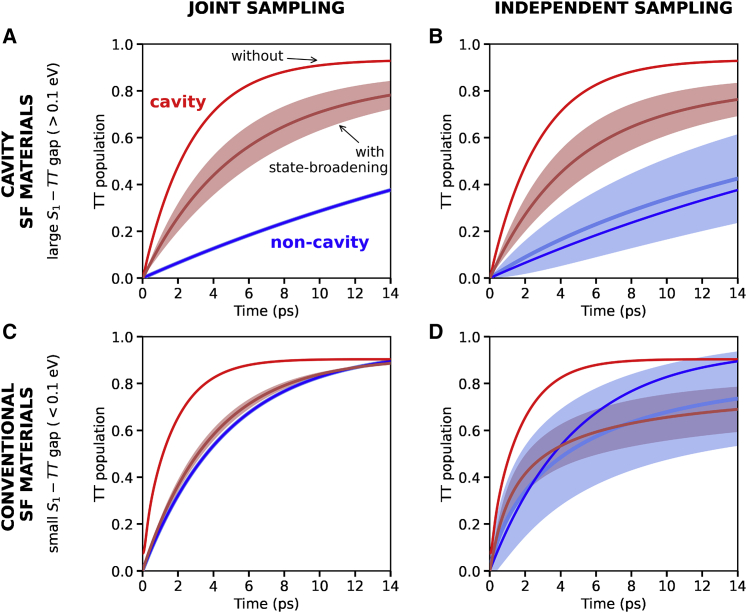
TT (diabatic) population dynamics with (light) and without (dark) state broadening (A–D) Non-cavity results are plotted in blue and strong coupling ones in red. The shaded area includes the mean ± SD of 50 realizations. Results are shown for the N=20 case. Coupling strength in (A) and (B) $g_{S_{1}}=42$ meV, and in (C) and (D) $g_{S_{1}}=17$ meV. The initial state for the cavity simulations is the LP. In the presence of state broadening, it is taken to be $\left|LP\right\rangle =c_{r}\left|r\right\rangle$, where $c_{r}$ is the cavity photon coefficient of the *r*-th eigenstate |r⟩, and only those eigenstates below the cavity photon are considered. For the non-cavity simulations, the initial state is taken as the eigenstate with the largest $S_{1}$ amplitude.

In Figure 5, we plot the strong coupling results (red) and compare them with the non-cavity ones (blue) with (light) and without (dark) state broadening. Figures 5A and 5B show the results for a system with an $S_{1}-TT$ gap of 0.2 eV, representative of the class of materials we propose for cavity-mediated singlet fission. Like in the previous sections, the coupling strength is chosen such that the LP is close to resonance with the TT eigenstates (${\text{Ω}}_{R}=375$ meV). In general, the singlet fission dynamics in the cavity is slowed down once state broadening is considered (light versus dark), but it is still much faster than the non-cavity situation (red versus blue). Therefore, our proposal is still operative in a realistic situation where the states have a finite width, and strong coupling can enhance singlet fission dynamics of materials that present a large $S_{1}-TT$ gap even in the presence of state broadening.

We explore next the situation in which the $S_{1}-TT$ gap is smaller (0.08 eV) and, accordingly, the Rabi splitting is also reduced (0.15 eV) to match the LP spectral location with that of the TT eigenstate. Our results are shown in Figures 5C and 5D for the two sampling cases. Note first that, when comparing the dark lines (the same in both panels), in which state broadening is not taken into account, the cavity and non-cavity dynamics (red versus blue) are more alike than for the case with a larger $S_{1}-TT$ gap (Figures 5A and 5B). As discussed before, this is due to the rapid relaxation to the dark-state manifold when the $S_{1}-TT$ gap is small. Moreover, when state broadening is incorporated in the simulations, cavity dynamics (light red line) turns out to be very similar to the non-cavity situation (light blue line) and the shaded areas even overlap. These results provide insight as to why, in a recent experimental work, no significant differences were found when comparing the cavity and non-cavity singlet fission dynamics of TIPS-pentacene.^37^ In the experimental setup, both the Rabi splitting and the FWHM of the $S_{1}$ vibronic peak that is coupled to the cavity mode were ∼0.1 eV. Notably, the $S_{1}-TT$ gap in TIPS-pentacene is also expected to be close to this value.^90^ Therefore, the experimental conditions resemble those illustrated in Figures 5C and 5D, which predict very moderate changes in singlet fission dynamics. This further confirms that singlet fission rates in slightly exothermic systems are not expected to be largely boosted by strong coupling. We also note that, although the cavity lifetime of the experimental work was much shorter that the one we consider here (∼ 20 fs versus 50 ps), the fact that singlet fission dynamics inside and outside the cavity took place at practically the same rate is an indication that, in the cavity experiments, the polaritons populated the dark-state manifold before decaying due to their photonic contribution. Therefore, the cavity lifetime is not a relevant parameter since it did not play an active role.

Finally, regarding the 50-ps cavity lifetime we consider in this work, although a long cavity lifetime is associated with a narrow linewidth, under strong coupling with organic molecules, when the polariton overlaps with (the tail of) the broad absorption band of the molecules, its effective linewidth will be increased, while decay of the excitation will not. We thus expect that even very long-lived cavity modes will lead to relatively broadband polaritons that can capture a sufficient fraction of the incoming light to be potentially useful for singlet fission photovoltaic applications.

To conclude, we have demonstrated that singlet fission dynamics can be accelerated under collective strong coupling via state mixing when the LP is almost resonant with the TT state. We have also shown that this effect is much more beneficial for compounds with large $S_{1}-TT$ gaps, thus reducing both the population transfer from the LP to the $S_{1}$ dark-state manifold and the detrimental impact that energetic broadening has on the resonant mechanism behind the rate acceleration. Given that the main characteristic of conventional singlet fission materials is a small $S_{1}-TT$ gap, and that systems such as polycyclic aromatic hydrocarbons are unstable and pose significant challenges for practical applications, our results provide a new perspective and suggest a new paradigm for cavity-mediated singlet fission with materials that have not previously been considered for singlet fission. Our results also highlight the fact that cavities with long-lived photons beyond a picosecond lifetime are required for singlet fission to benefit from strong light-matter coupling. We hope that our results can serve as a guide and inspire future experiments to realize efficient singlet fission with unconventional compounds in optical cavities.

## Experimental procedures

### Resource availability

#### Lead contact

Further information and requests for resources should be directed to and will be fulfilled by the lead contact, Clàudia Climent (ccliment@sas.upenn.edu).

#### Materials availability

This study did not generate new unique materials.

### Methods

In our simulations, each molecular excited state $\left\{\left|TT_{k}\right\rangle ,\left|S_{1_{k}}\right\rangle ,\left|CT_{k}\right\rangle \right\}$ is coupled to identical and independent baths. We consider Ohmic spectral densities with a Lorentizian cutoff $J\left(w\right)=2\lambda \text{Ω}w/\left(w^{2}+{\text{Ω}}^{2}\right)$, where Ω is the cutoff frequency and λ the reorganization energy. Following Berkelbach et al.,^69^ we take the values Ω=150 meV and λ=25 meV, which are characteristic of organic aromatic molecules.

The power spectrum is given by

(Equation 10) $$S\left(w\right)=\left\{ \begin{array}{ll} 2J\left(w\right)\left(n\left(w,T\right)+1\right) & ,w>0\\ 4kT\lambda /\text{Ω} & ,w=0\\ 2J\left(-w\right)n\left(-w,T\right) & ,w<0 \end{array}\right.$$

with the Bose occupation factor $n\left(w,T\right)={\left(e^{w/k_{B}T}-1\right)}^{-1}$, and we take T=300 K.

To account for the finitie cavity lifetime, we include a Lindblad term in our simulations, $\frac{\kappa }{2}L_{\hat{a}}\left[\hat{\rho }\right]$, where $L_{\hat{a}}=2\hat{a}\hat{\rho }{\hat{a}}^{†}-\left\{\hat{\rho },{\hat{a}}^{†}\hat{a}\right\}$, where $\hat{a}$ is the bosonic destruction operator of the cavity photon, and κ is the lifetime.

Note that we do not rely on the secular approximation of the Bloch-Redfield equation. This is because this approximation fails when there are eigenstates close in energy. The master equation has been solved with the Qutip package.^91^^,^^92^

## Data Availability

The datasets supporting the current study are available from the lead contact upon reasonable request.

## References

[1] Ebbesen T.W. (2016). Hybrid light-matter states in a molecular and material science perspective. Acc. Chem. Res..

[2] Feist J., Galego J., Garcia-Vidal F.J. (2018). Polaritonic chemistry with organic molecules. ACS Photonics.

[3] Garcia-Vidal F.J., Ciuti C., Ebbesen T.W. (2021). Manipulating matter by strong coupling to vacuum fields. Science.

[4] Herrera F., Owrutsky J. (2020). Molecular polaritons for controlling chemistry with quantum optics. J. Chem. Phys..

[5] Hertzog M., Wang M., Mony J., Börjesson K. (2019). Strong light–matter interactions: a new direction within chemistry. Chem. Soc. Rev..

[6] Ribeiro R.F., Martínez-Martínez L.A., Du M., Campos-Gonzalez-Angulo J., Yuen-Zhou J. (2018). Polariton chemistry: controlling molecular dynamics with optical cavities. Chem. Sci..

[7] Galego J., Garcia-Vidal F.J., Feist J. (2015). Cavity-induced modifications of molecular structure in the strong-coupling regime. Phys. Rev. X.

[8] Hutchison J.A., Schwartz T., Genet C., Devaux E., Ebbesen T.W. (2012). Modifying chemical landscapes by coupling to vacuum fields. Angew. Chem. Int. Edition.

[9] Galego J., Garcia-Vidal F.J., Feist J. (2016). Suppressing photochemical reactions with quantized light fields. Nat. Commun..

[10] Galego J., Garcia-Vidal F.J., Feist J. (2017). Many-molecule reaction triggered by a single photon in polaritonic chemistry. Phys. Rev. Lett..

[11] Cederbaum L.S. (2021). Polaritonic states of matter in a rotating cavity. J. Phys. Chem. Lett..

[12] Csehi A., Kowalewski M., Halász G.J., Vibók Á. (2019). Ultrafast dynamics in the vicinity of quantum light-induced conical intersections. New J. Phys..

[13] Kowalewski M., Bennett K., Mukamel S. (2016). Cavity femtochemistry: manipulating nonadiabatic dynamics at avoided crossings. J. Phys. Chem. Lett..

[14] Ulusoy I.S., Gomez J.A., Vendrell O. (2019). Modifying the nonradiative decay dynamics through conical intersections via collective coupling to a cavity mode. J. Phys. Chem. A.

[15] Vendrell O. (2018). Collective Jahn-Teller interactions through light-matter coupling in a cavity. Phys. Rev. Lett..

[16] Coles D.M., Somaschi N., Michetti P., Clark C., Lagoudakis P.G., Savvidis P.G., Lidzey D.G. (2014). Polariton-mediated energy transfer between organic dyes in a strongly coupled optical microcavity. Nat. Mater..

[17] Du M., Martínez-Martínez L.A., Ribeiro R.F., Hu Z., Menon V.M., Yuen-Zhou J. (2018). Theory for polariton-assisted remote energy transfer. Chem. Sci..

[18] Herrera F., Spano F.C. (2016). Cavity-controlled chemistry in molecular ensembles. Phys. Rev. Lett..

[19] Mauro L., Caicedo K., Jonusauskas G., Avriller R. (2021). Charge-transfer chemical reactions in nanofluidic Fabry-Pérot cavities. Phys. Rev. B.

[20] Sáez-Blázquez R., Feist J., Fernández-Domínguez A.I., García-Vidal F.J. (2018). Organic polaritons enable local vibrations to drive long-range energy transfer. Phys. Rev. B.

[21] Semenov A., Nitzan A. (2019). Electron transfer in confined electromagnetic fields. J. Chem. Phys..

[22] Zhong X., Chervy T., Wang S., George J., Thomas A., Hutchison J.A., Devaux E., Genet C., Ebbesen T.W. (2016). Non-radiative energy transfer mediated by hybrid light-matter states. Angew. Chem. Int. Ed..

[23] Antoniou P., Suchanek F., Varner J.F., Foley J.J. (2020). Role of cavity losses on nonadiabatic couplings and dynamics in polaritonic chemistry. J. Phys. Chem. Lett..

[24] Davidsson E., Kowalewski M. (2020). Simulating photodissociation reactions in bad cavities with the Lindblad equation. J. Chem. Phys..

[25] Felicetti S., Fregoni J., Schnappinger T., Reiter S., de Vivie-Riedle R., Feist J. (2020). Photoprotecting uracil by coupling with lossy nanocavities. J. Phys. Chem. Lett..

[26] Ulusoy I.S., Vendrell O. (2020). Dynamics and spectroscopy of molecular ensembles in a lossy microcavity. J. Chem. Phys..

[27] Agranovich V.M., Litinskaia M., Lidzey D.G. (2003). Cavity polaritons in microcavities containing disordered organic semiconductors. Phys. Rev. B.

[28] Groenhof G., Climent C., Feist J., Morozov D., Toppari J.J. (2019). Tracking polariton relaxation with multiscale molecular dynamics simulations. J. Phys. Chem. Lett..

[29] Herrera F., Spano F.C. (2017). Absorption and photoluminescence in organic cavity QED. Phys. Rev. A..

[30] Herrera F., Spano F.C. (2017). Dark vibronic polaritons and the spectroscopy of organic microcavities. Phys. Rev. Lett..

[31] Lidzey D.G., Fox A.M., Rahn M.D., Skolnick M.S., Agranovich V.M., Walker S. (2002). Experimental study of light emission from strongly coupled organic semiconductor microcavities following nonresonant laser excitation. Phys. Rev. B.

[32] Litinskaya M., Reineker P., Agranovich V.M. (2004). Fast polariton relaxation in strongly coupled organic microcavities. J. Lumin..

[33] Mony J., Climent C., Petersen A.U., Moth-Poulsen K., Feist J., Börjesson K. (2021). Photoisomerization efficiency of a solar thermal fuel in the strong coupling regime. Adv. Funct. Mater..

[34] Tichauer R.H., Feist J., Groenhof G. (2021). Multi-scale dynamics simulations of molecular polaritons: the effect of multiple cavity modes on polariton relaxation. J. Chem. Phys..

[35] Virgili T., Coles D., Adawi A.M., Clark C., Michetti P., Rajendran S.K., Brida D., Polli D., Cerullo G., Lidzey D.G. (2011). Ultrafast polariton relaxation dynamics in an organic semiconductor microcavity. Phys. Rev. B.

[36] Eizner E., Martínez-Martínez L.A., Yuen-Zhou J., Kéna-Cohen S. (2019). Inverting singlet and triplet excited states using strong light-matter coupling. Sci. Adv..

[37] Liu B., Menon V.M., Sfeir M.Y. (2020). The role of long-lived excitons in the dynamics of strongly coupled molecular polaritons. ACS Photonics.

[38] Vurgaftman I., Simpkins B.S., Dunkelberger A.D., Owrutsky J.C. (2020). Negligible effect of vibrational polaritons on chemical reaction rates via the density of states pathway. J. Phys. Chem. Lett..

[39] Stranius K., Herzog M., Börjesson K. (2018). Selective manipulation of electronically excited states through strong light-matter interactions. Nat. Comm..

[40] Yu Y., Mallick S., Wang M., Börjesson K. (2021). Barrier-free reverse-intersystem crossing in organic molecules by strong light-matter coupling. Nat. Commun..

[41] Martínez-Martínez L.A., Eizner E., Kéna-Cohen S., Yuen-Zhou J. (2019). Triplet harvesting in the polaritonic regime: a variational polaron approach. J. Chem. Phys..

[42] Martínez-Martínez L.A., Du M., Ribeiro R.F., Kéna-Cohen S., Yuen-Zhou J. (2018). Polariton-assisted singlet fission in acene aggregates. J. Phys. Chem. Lett..

[43] del Pino J., Feist J., Garcia-Vidal F.J. (2015). Quantum theory of collective strong coupling of molecular vibrations with a microcavity mode. New J. Phys..

[44] Casanova D. (2018). Theoretical modeling of singlet fission. Chem. Rev..

[45] Smith M.B., Michl J. (2010). Singlet fission. Chem. Rev..

[46] Smith M.B., Michl J. (2013). Recent advances in singlet fission. Annu. Rev. Phys. Chem..

[47] Shockley W., Queisser H.J. (1961). Detailed balance limit of efficiency of p-n junction solar cells. J. Appl. Phys..

[48] Congreve D.N., Lee J., Thompson N.J., Hontz E., Yost S.R., Reusswig P.D., Bahlke M.E., Reineke S., Van Voorhis T., Baldo M.A. (2013). External quantum efficiency above 100% in a singlet-exciton-fission-based organic photovoltaic cell. Science.

[49] Einzinger M., Wu T., Kompalla J.F., Smith H.L., Perkinson C.F., Nienhaus L., Wieghold S., Congreve D.N., Kahn A., Bawendi M.G., Baldo M.A. (2019). Sensitization of silicon by singlet exciton fission in tetracene. Nature.

[50] Hanna M.C., Nozik A.J. (2006). Solar conversion efficiency of photovoltaic and photoelectrolysis cells with carrier multiplication absorbers. J. Appl. Phys..

[51] Miyata K., Conrad-Burton F.S., Geyer F.L., Zhu X.-Y. (2019). Triplet pair states in singlet fission. Chem. Rev..

[52] Sanders S.N., Pun A.B., Parenti K.R., Kumarasamy E., Yablon L.M., Sfeir M.Y., Campos L.M. (2019). Understanding the bound triplet-pair state in singlet fission. Chem.

[53] Gu B., Mukamel S. (2021). Optical-cavity manipulation of conical intersections and singlet fission in pentacene dimers. J. Phys. Chem. Lett..

[54] Takahashi S., Watanabe K., Matsumoto Y. (2019). Singlet fission of amorphous rubrene modulated by polariton formation. J. Chem. Phys..

[55] Zhang B., Zhao Y., Liang W. (2021). Joint effects of exciton–exciton and exciton–photon couplings on the singlet fission dynamics in organic aggregates. J. Phys. Chem. C.

[56] Chikkaraddy R., de Nijs B., Benz F., Barrow S.J., Scherman O.A., Rosta E., Demetriadou A., Fox P., Hess O., Baumberg J.J. (2016). Single-molecule strong coupling at room temperature in plasmonic nanocavities. Nature.

[57] Eizner E., Brodeur J., Barachati F., Sridharan A., Kéna-Cohen S. (2018). Organic photodiodes with an extended responsivity using ultrastrong light–matter coupling. ACS Photonics.

[58] Esteso V., Caliò L., Espinós H., Lavarda G., Torres T., Feist J., García-Vidal F.J., Bottari G., Míguez H. (2021). Light-harvesting properties of a subphthalocyanine solar absorber coupled to an optical cavity. Solar RRL.

[59] Nikolis V.C., Mischok A., Siegmund B., Kublitski J., Jia X., Benduhn J., Hörmann U., Neher D., Gather M.C., Spoltore D., Vandewal K. (2019). Strong light-matter coupling for reduced photon energy losses in organic photovoltaics. Nat. Commun..

[60] Sáez-Blázquez R., Feist J., Romero E., Fernández-Domínguez A.I., García-Vidal F.J. (2019). Cavity-modified exciton dynamics in photosynthetic units. J. Phys. Chem. Lett..

[61] Berghuis A.M., Halpin A., Le-Van Q., Ramezani M., Wang S., Murai S., Gómez Rivas J. (2019). Enhanced delayed fluorescence in tetracene crystals by strong light-matter coupling. Adv. Funct. Mater..

[62] Polak D., Jayaprakash R., Lyons T.P., Martínez-Martínez L.Á., Leventis A., Fallon K.J., Coulthard H., Bossanyi D.G., Georgiou K., Petty A.J. (2020). Manipulating molecules with strong coupling: harvesting triplet excitons in organic exciton microcavities. Chem. Sci..

[63] Ye C., Mallick S., Hertzog M., Kowalewski M., Börjesson K. (2021). Direct transition from triplet excitons to hybrid light–matter states via triplet–triplet annihilation. J. Am. Chem. Soc..

[64] Ambrosio F., Troisi A. (2014). Singlet fission in linear chains of molecules. J. Chem. Phys..

[65] Feng X., Kolomeisky A.B., Krylov A.I. (2014). Dissecting the effect of morphology on the rates of singlet fission: insights from theory. The J. Phys. Chem. C.

[66] Matsika S., Feng X., Luzanov A.V., Krylov A.I. (2014). What we can learn from the norms of one-particle density matrices, and what we can’t: some results for interstate properties in model singlet fission systems. The J. Phys. Chem. A.

[67] Teichen P.E., Eaves J.D. (2015). Collective aspects of singlet fission in molecular crystals. J. Chem. Phys..

[68] Yost S.R., Lee J., Wilson M.W.B., Wu T., McMahon D.P., Parkhurst R.R., Thompson N.J., Congreve D.N., Rao A., Johnson K. (2014). A transferable model for singlet-fission kinetics. Nat. Chem..

[69] Berkelbach T.C., Hybertsen M.S., Reichman D.R. (2013). Microscopic theory of singlet exciton fission. I. General formulation. J. Chem. Phys..

[70] Berkelbach T.C., Hybertsen M.S., Reichman D.R. (2013). Microscopic theory of singlet exciton fission. II. Application to pentacene dimers and the role of superexchange. J. Chem. Phys..

[71] Mirjani F., Renaud N., Gorczak N., Grozema F.C. (2014). Theoretical investigation of singlet fission in molecular dimers: the role of charge transfer states and quantum interference. J. Phys. Chem. C.

[72] Jaynes E.T., Cummings F.W. (1963). Comparison of quantum and semiclassical radiation theories with to the beam maser. Proc. IEEE.

[73] Fait J., Putz S., Wachter G., Schalko J., Schmid U., Arndt M., Trupke M. (2021). High finesse microcavities in the optical telecom O-band. Appl. Phys. Lett..

[74] Najer D., Renggli M., Riedel D., Starosielec S., Warburton R.J. (2017). Fabrication of mirror templates in silica with micron-sized radii of curvature. Appl. Phys. Lett..

[75] Wilson M.W.B., Rao A., Clark J., Kumar R.S.S., Brida D., Cerullo G., Friend R.H. (2011). Ultrafast dynamics of exciton fission in polycrystalline pentacene. J. Am. Chem. Soc..

[76] Tavis M., Cummings F.W. (1968). Exact solution for an N-Molecule-Radiation-Field Hamiltonian. Phys. Rev..

[77] Tavis M., Cummings F.W. (1969). Approximate solutions for an N-molecule radiation-field Hamiltonian. Phys. Rev..

[78] Ly J.T., Lopez S.A., Lin J.B., Kim J.J., Lee H., Burnett E.K., Zhang L., Aspuru-Guzik A., Houk K.N., Briseno A.L. (2018). Oxidation of rubrene, and implications for device stability. J. Mater. Chem. C.

[79] Maliakal A., Raghavachari K., Katz H., Chandross E., Siegrist T. (2004). Photochemical stability of pentacene and a substituted pentacene in solution and in thin films. Chem. Mater..

[80] Mondal R., Tönshoff C., Khon D., Neckers D.C., Bettinger H.F. (2009). Synthesis, stability, and photochemistry of pentacene, hexacene, and heptacene: a matrix isolation study. J. Am. Chem. Soc..

[81] Sanders S.N., Kumarasamy E., Fallon K.J., Sfeir M.Y., Campos L.M. (2020). Singlet fission in a hexacene dimer: energetics dictate dynamics. Chem. Sci..

[82] Zhang Y.-D., Wu Y., Xu Y., Wang Q., Liu K., Chen J.-W., Cao J.-J., Zhang C., Fu H., Zhang H.-L. (2016). Excessive exoergicity reduces singlet exciton fission efficiency of heteroacenes in solutions. J. Am. Chem. Soc..

[83] Groom C.R., Bruno I.J., Lightfoot M.P., Ward S.C. (2016). The Cambridge structural Database. Acta Crystallogr. Section B.

[84] Padula D., Omar Ö.H., Nematiaram T., Troisi A. (2019). Singlet fission molecules among known compounds: finding a few needles in a haystack. Energy Environ. Sci..

[85] Ern V., McGhie A.R. (1971). Quenching of triplet excitons in anthracene crystals by internal beta-irradiation. Mol. Crystals Liquid Crystals.

[86] Ern V., Merrifield R.E. (1968). Magnetic field effect on triplet exciton quenching in organic crystals. Phys. Rev. Lett..

[87] Kéna-Cohen S., Maier S.A., Bradley D.D.C. (2013). Ultrastrongly coupled exciton–polaritons in metal-clad organic semiconductor microcavities. Adv. Opt. Mater..

[88] Montalti M., Credi A., Prodi L., Gandolfi M.T. (2006).

[89] Burdett J.J., Müller A.M., Gosztola D., Bardeen C.J. (2010). Excited state dynamics in solid and monomeric tetracene: the roles of superradiance and exciton fission. J. Chem. Phys..

[90] Tamura H., Huix-Rotllant M., Burghardt I., Olivier Y., Beljonne D. (2015). First-principles quantum dynamics of singlet fission: coherent versus thermally activated mechanisms governed by molecular π stacking. Phys. Rev. Lett..

[91] Johansson J., Nation P., Nori F. (2012). Qutip: an open-source python framework for the dynamics of open quantum systems. Computer Phys. Commun..

[92] Johansson J., Nation P., Nori F. (2013). Qutip 2: a python framework for the dynamics of open quantum systems. Computer Phys. Commun..

